# Author Correction: MiR-497∼195 cluster regulates angiogenesis during coupling with osteogenesis by maintaining endothelial Notch and HIF-1α activity

**DOI:** 10.1038/s41467-025-60624-5

**Published:** 2025-06-23

**Authors:** Mi Yang, Chang-Jun Li, Xi Sun, Qi Guo, Ye Xiao, Tian Su, Man-Li Tu, Hui Peng, Qiong Lu, Qing Liu, Hong-Bo He, Tie-Jian Jiang, Min-Xiang Lei, Mei Wan, Xu Cao, Xiang-Hang Luo

**Affiliations:** 1 Department of Endocrinology, Endocrinology Research Center, Xiangya Hospital of Central South University, Changsha, Hunan 410008 China; 2 Department of Orthopaedic Surgery, Johns Hopkins University School of Medicine, Baltimore, Maryland 21205 USA; 3 Department of Endocrinology, The Second Xiangya Hospital of Central South University, Changsha, Hunan 410011 China; 4 Key Laboratory of Organ Injury, Aging and Regenerative Medicine of Hunan Province, Changsha, 410008 China; 5 Department of Orthopedic Surgery, Xiangya Hospital, Central South University, Changsha, 410008 China

Correction to: *Nature Communications* 10.1038/ncomms16003, published online 07 July 2017

In the version of this article initially published, due to figure preparation mistakes, the images shown as Supplementary Fig. 5a, WT: 12mo and Supplementary Fig. 5c, WT: 3mo were from incorrect sources. The figure presented as Fig. 1, below, serves to correct the article.

Fig. 1 | Corrected Supplementary Fig. 5
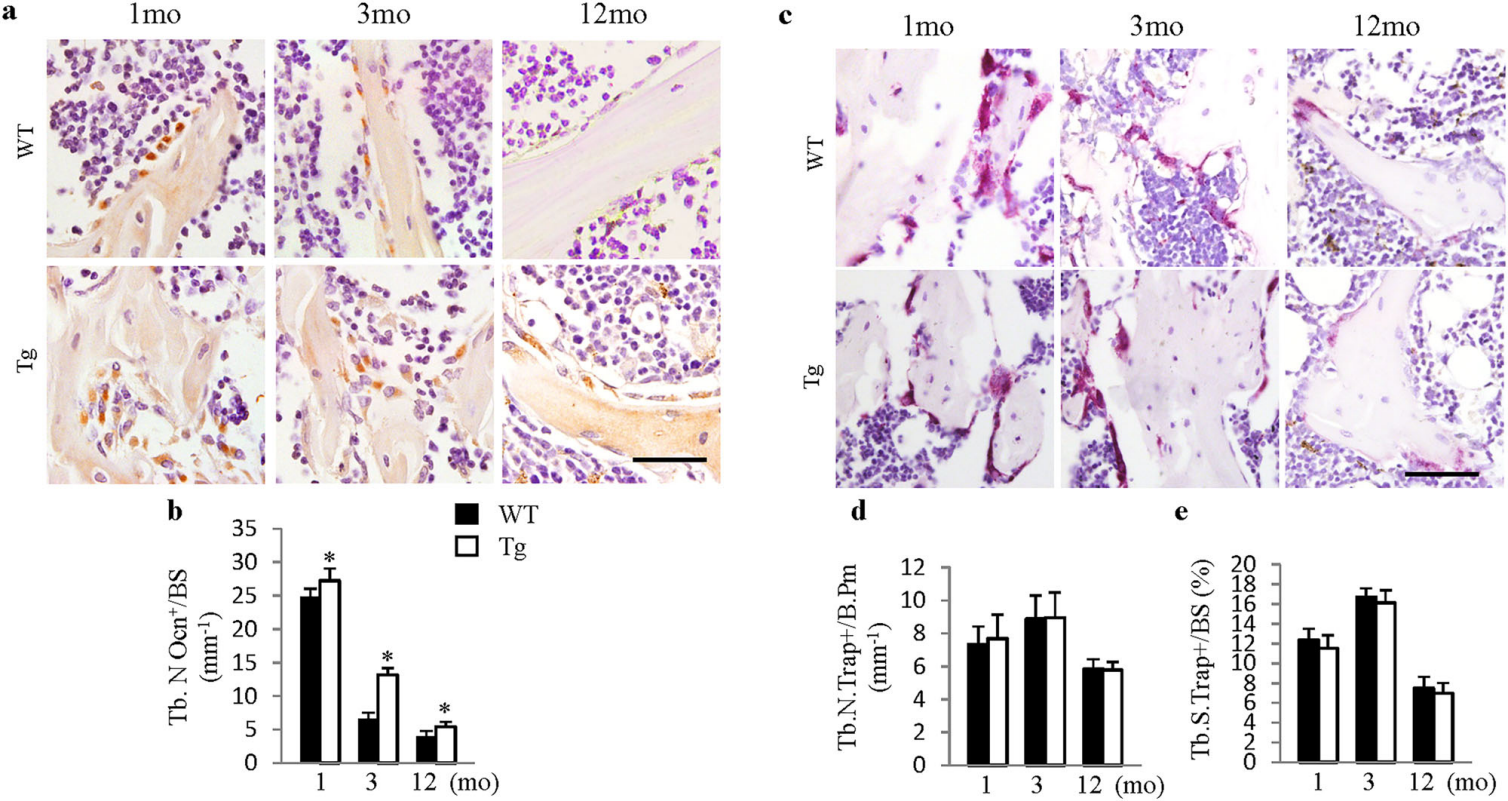


## Supplementary information

Corrected Supplementary Fig. 5

