# Supplementary material for: MiR-497∼195 cluster regulates angiogenesis during coupling with osteogenesis by maintaining endothelial Notch and HIF-1α activity
Source: Nat Commun. 2017 Jul 7;8:16003. doi: 10.1038/ncomms16003 (PMC5504303; doi:10.1038/ncomms16003)
Supplement: Supplementary Information [file ncomms16003-s1.pdf]

Type of file: PDF
Title of file for HTML: Supplementary Information
Description: Supplementary Figures and Supplementary Tables.

Type of file: PDF
Title of file for HTML: Peer Review File
Description:

Supplementary Figure 1

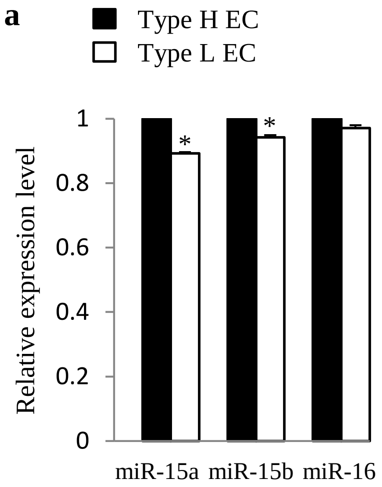

Supplementary Figure 1. Expression level of miR-15a, miR-15b and miR-16 in isolated endothelial cells.

(**a**) qRT-PCR analysis of miR-15a, miR-15b and miR-16 level in Type H ECs and Type L ECs. n=5 in each group from three independent experiments. Data shown as mean ± SD. **P* < 0.05, (Student's *t*-test).

Supplementary Figure 2

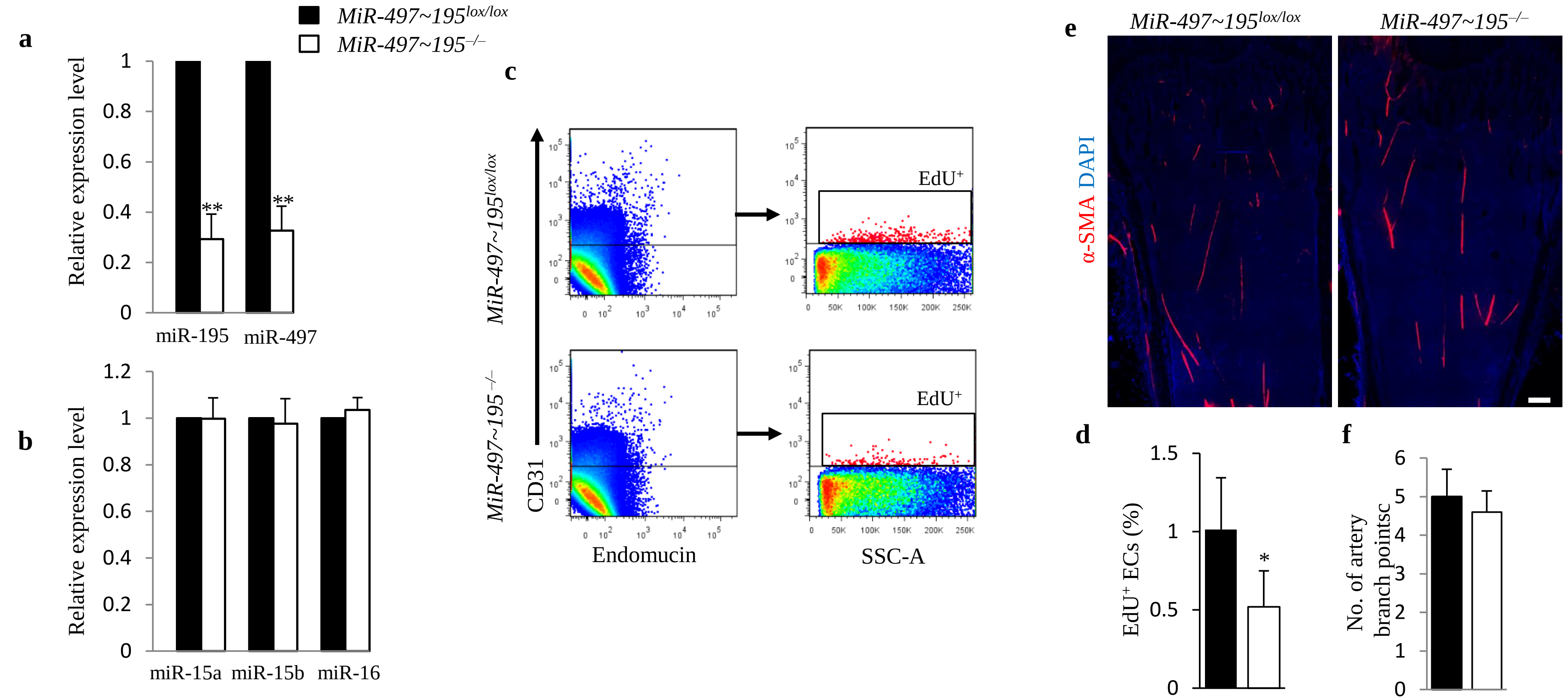

Supplementary Figure 2. Mir-15 family Expression and endothelial cells proliferation and artery formation in endothelial cell-specific miR-497~195 knockout mice .

(**a**) Expression level of miR-195/miR-497 in endothelial cells from 3 month-old *MiR-497~195*$^{-/-}$ mice and *MiR-497~195*$^{lox/lox}$ control. (**b**) qRT-PCR analysis of miR-15a, miR-15b and miR-16 level in endothelial cells from 3 month-old *MiR-497~195*$^{-/-}$ mice and *MiR-497~195*$^{lox/lox}$ control. (**c-d**) FACS analysis dot plot (**c**) and quantification (**d**) of EdU labeled endothelial cells (defined as $CD31^{+}CD45^{-}$) in BM of one-month-old *MiR-497~195*$^{-/-}$ mice and *MiR-497~195*$^{lox/lox}$ control. (**e**) Representative images of α-SMA (red) immunostaining in thick sections (80 μm) of one-month-old *MiR-497~195*$^{-/-}$ mice and *MiR-497~195*$^{lox/lox}$ control. Nuclei, DAPI (blue). Scale bar: 200μm. (**f**) Quantitation of α-SMA^{+} artery. n=5 in each group from three independent experiments. Data shown as mean ± SD. *$P < 0.05$, **$P<0.01$, (Student's *t*-test).

Supplementary Figure 3

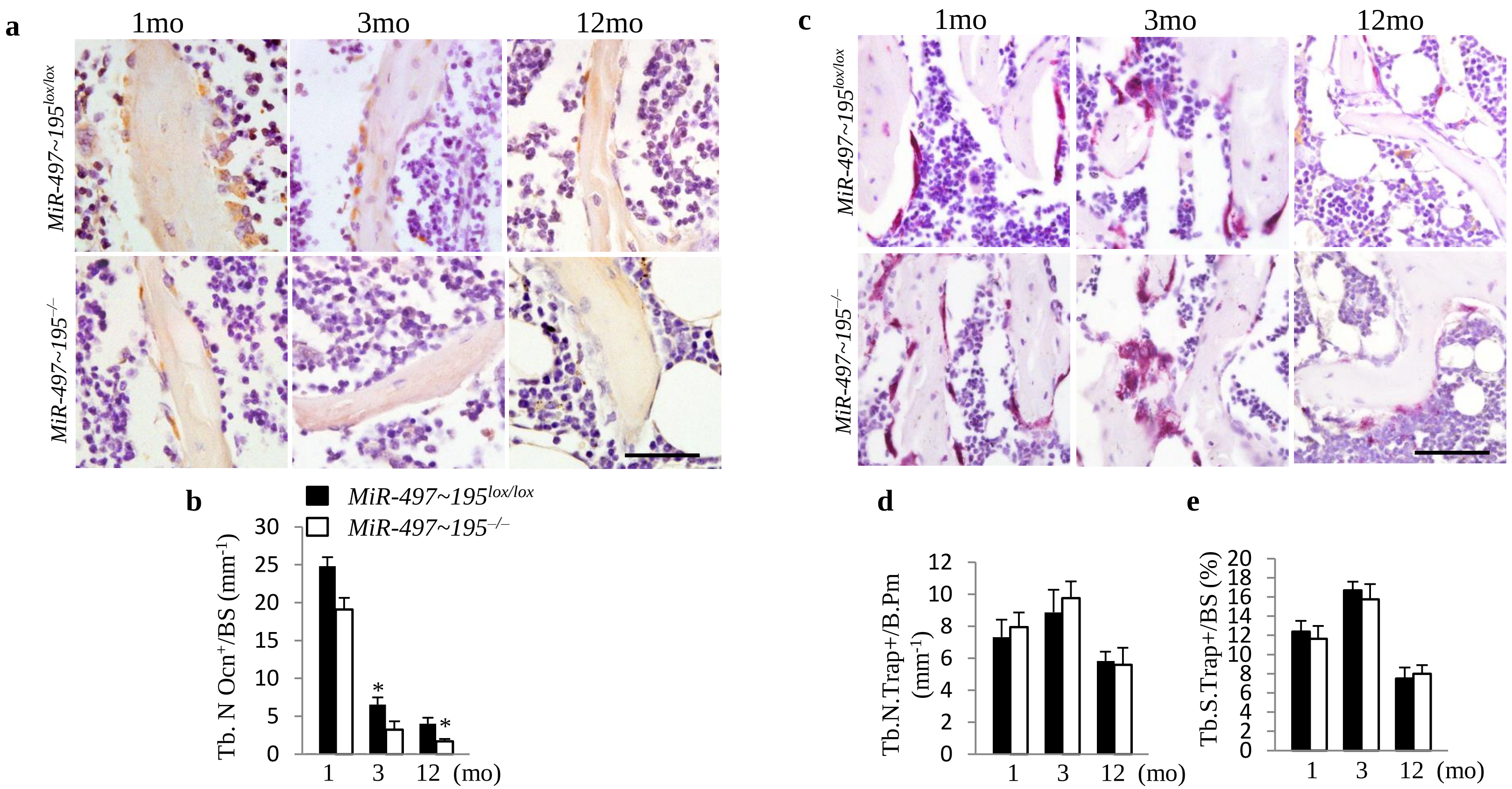

Supplementary Figure 3. Decreased number of osteoblast cells and BMSCs osteogenic differentiation capacity in endothelial cell-specific miR-497~195 knockout mice.

(**a**) Representative images of osteocalcin staining from different time point endothelial cell-specific miR-497~195 knockout mice (*MiR-497~195*$^{-/-}$) and their control (*MiR-497~195*$^{lox/lox}$) with quantification of number of osteoblasts (**b**) in distal femora. (**c**) Representative images of TRAP staining of femora from different time point *MiR-497~195*$^{-/-}$ mice and their control. (**d-e**) Quantification data of TRAP^{+} cells in trabecular bone surface. Number of TRAP^{+} cells per bone perimeter (Tb.N.Trap^{+}/B.Pm), and TRAP^{+} cells surface per bone surface (Tb.S.Trap^{+}/BS), were measured. Scale bar: 50 μm. n=5 mice in each group from three independent experiments. Data shown as mean ± SD. *$P < 0.05$, (Student's *t*-test).

Supplementary Figure 4

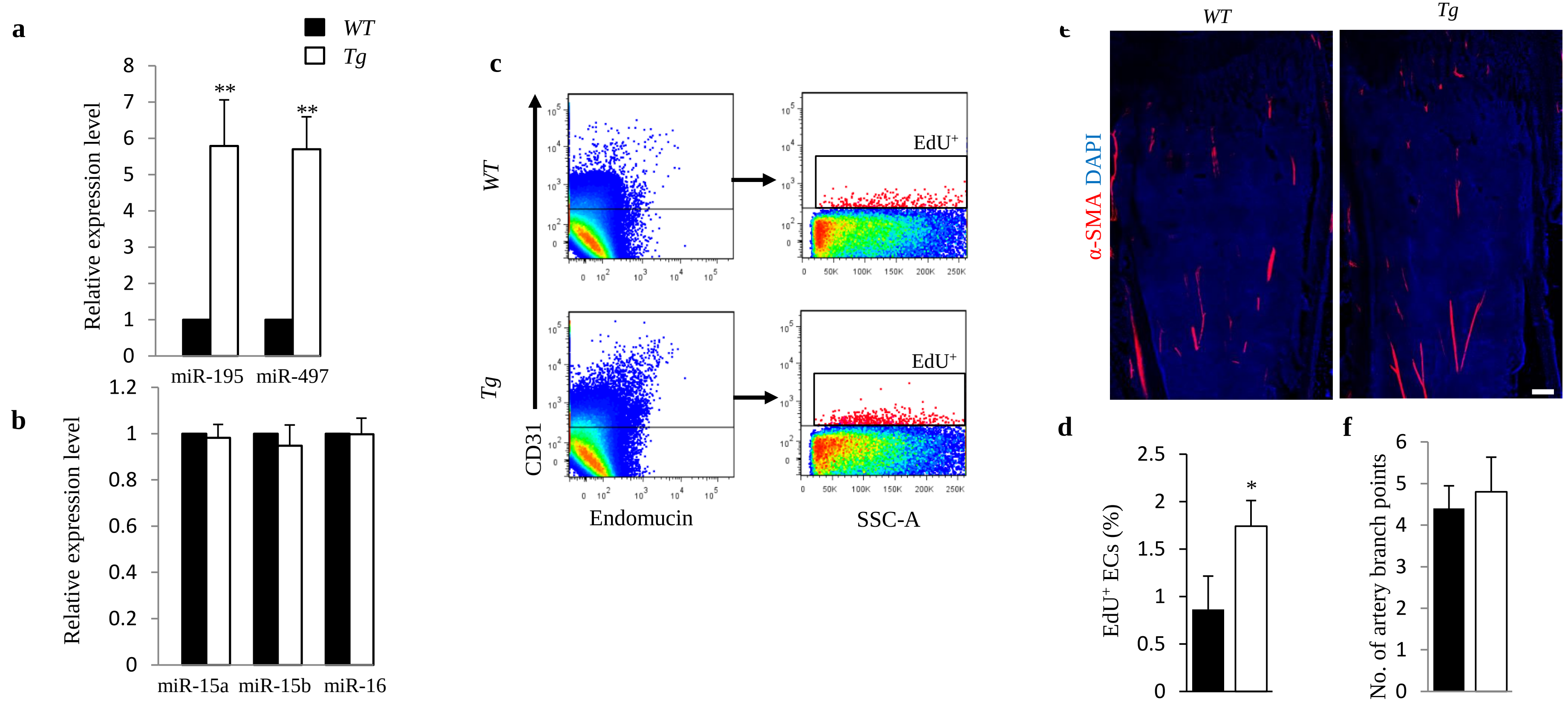

Supplementary Figure 4. Mir-15 family Expression and endothelial cells proliferation and artery formation in endothelial cell-specific miR-497~195 overexpression mice .

(**a**) Expression of miR-195/miR-497 in endothelial cells from 3 month-old *WT, TG* mice was determined using qRT-PCR. (**b**) qRT-PCR analysis of miR-15a, miR-15b and miR-16 level in 3 month-old *TG* mice and *WT* control. (**c-d**) FACS analysis dot plot (**c**) and quantification (**d**) of EdU labeled endothelial cells (defined as $CD31^{+}CD45^{-}$) in BM of one-month-old *WT* and miR-497~195 transgenic (*Tg*) mice. (**e**) Representative images of α-SMA (red) immunostaining in thick sections (80 μm) of one-month-old *WT* and miR-497~195 transgenic (*Tg*) mice. Nuclei, DAPI (blue). Scale bar: 200μm. (**f**) Quantitation of α-SMA^{+} artery. n=5 in each group from three independent experiments. Data shown as mean ± SD. *P < 0.05 , **P<0.01, (Student's *t*-test).

Supplementary Figure 5

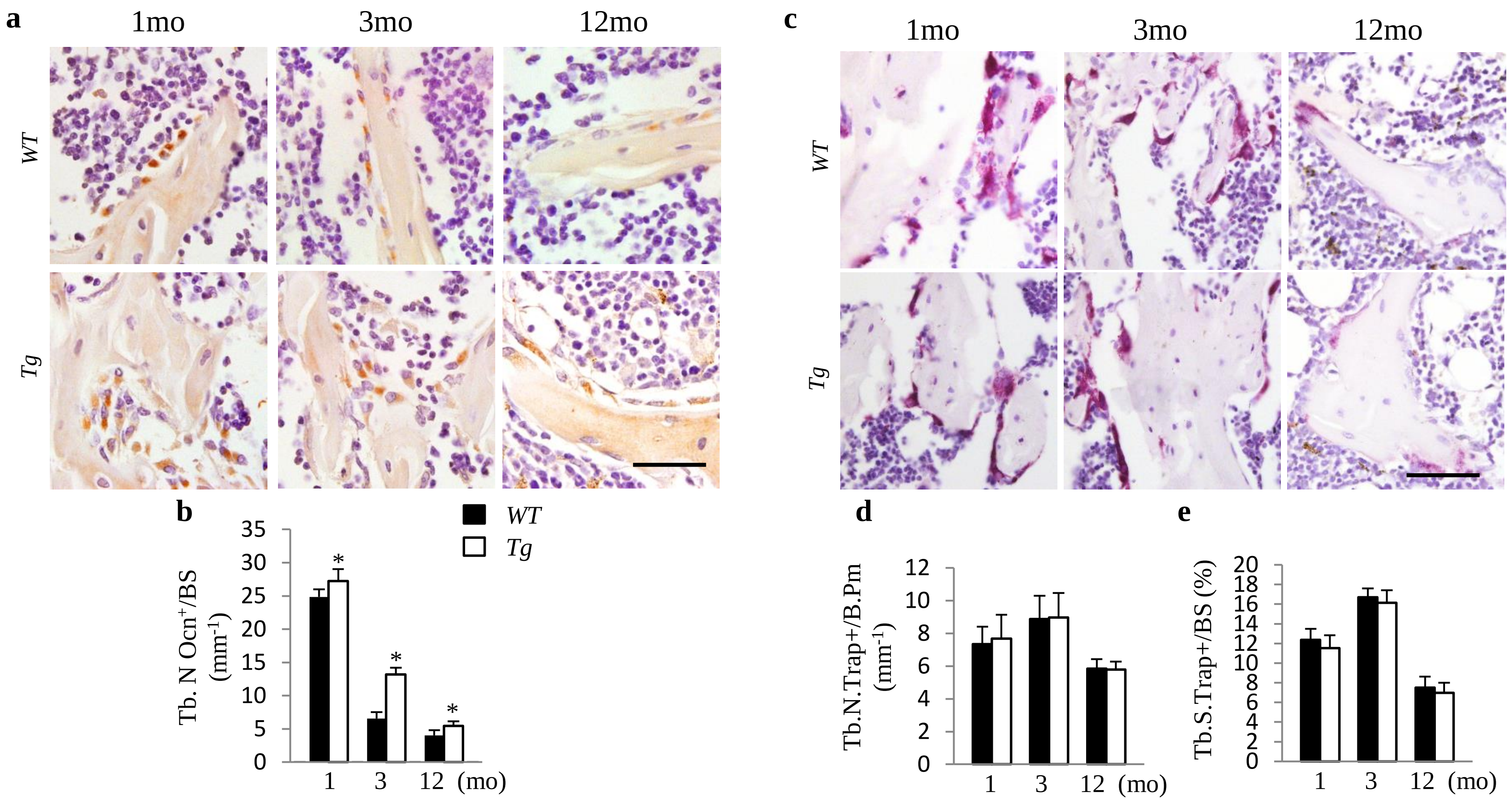

Supplementary Figure 5. Increased number of osteoblast cells and BMSCs osteogenic differentiation capacity in endothelial cell-specific miR-497~195 overexpression mice.

(**a**) Representative images of osteocalcin staining of femora from different time point endothelial cell-specific miR-497~195 transgenic mice (*Tg*) and their control (*WT*) with quantification of number of osteoblasts (**b**) in distal femora. (**c**) Representative images of TRAP staining of femora from different time point in *Tg* and *WT* mice. (**d-e**) Quantification data of $TRAP^+$ cells in trabecular bone surface. Number of $TRAP^+$ cells per bone perimeter ($Tb.N.Trap^+/B.Pm$), and $TRAP^+$ cells surface per bone surface ($Tb.S.Trap^+/BS$) were measured. Scale bar: 50 μm. n=5 in each group from three independent experiments. Data shown as mean ± SD. *$P < 0.05$, (Student's *t*-test).

Supplementary Figure 6

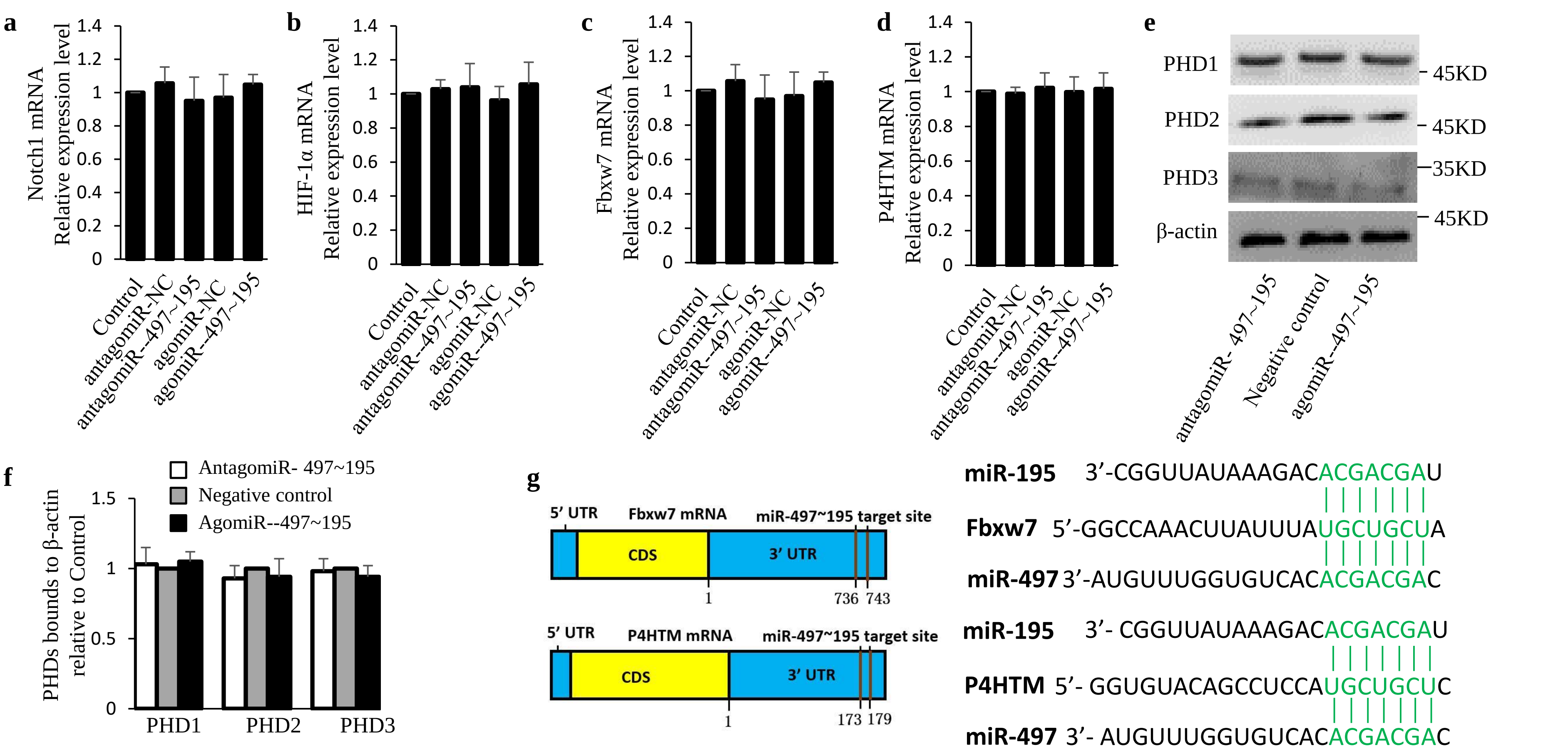

Supplementary Figure 6. MiR-497~195 cluster promotes CD31 and endomucin expression in cultured endothelial cells via targeting Fbxw7 and P4HTM and maintaining endothelial Notch and HIF-*α* activity. Bone marrow endothelial cells (BMECs) transfected with agomiR-497~195, antagomiR-497~195, or their negative controls. (**a-d**) qRT-PCR analysis of the relative levels of Notch1 (**a**), HIFα (**b**), Fbxw7 (**c**) and P4HTM (**d**) mRNA expression in endothelial cells. (**e-f**) Western blot analysis (**e**) and the quantitation (**f**) of the relative levels of PHD1-3 protein expression in BMECs transfected with agomiR-497~195, antagomiR-497~195 or control. (n=3 in each group from three independent experiments). (**g**) Schematic and target region of miR-497~195 putative target sites in mouse Fbxw7 and P4HTM 3′-UTR. NC, negative control. CDS, coding sequence**.** Data shown as mean ± SD. (analysis of variance (ANOVA)).

Supplementary Figure 7

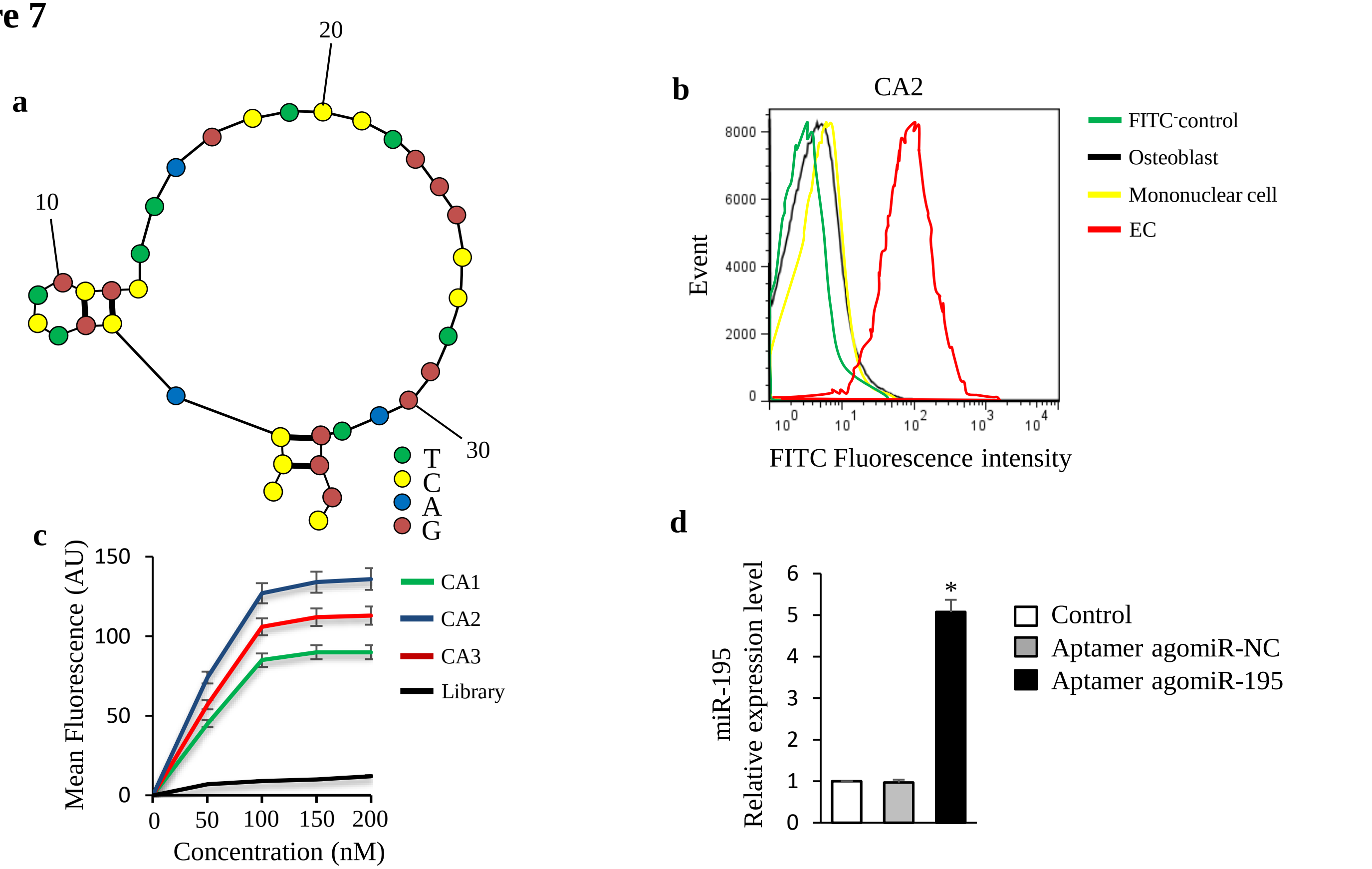

Supplementary Figure 7. Identification of endothelial cell-specific aptamer-agomiR-195. (**a**) Proposed secondary structure of EC aptamer. Thick lines, hydrogen bonds between base pairs; thin lines, main phosphodiester bond. (**b**) The red curve represents the ECs incubated with fluorescein isothiocyanate (FITC) aptamer; the black curve and yellow curve represent the osteoblasts and mononuclear cells incubated with FITC aptamer respectively, the green curve represents the FITC negative control. (**c**) Flow cytometry to determine the binding affinity of the candidate aptamers (CA1,CA2,CA3) for the EC cells at the concentrations ranged from 50 nM to 200 nM. (**d**) qRT-PCR analysis of the levels of miR-195 expression in ECs of mice with EC-specific angomiR-195 delivery. Aptamer-angomiR-195 was injected via tail vein of 12-month-old mice once per week for 3 months. NC, negative control. CA, candidate aptamer. n=5 mice in each group from three independent experiments. Data shown as mean ± SD. *P < 0.05, (analysis of variance (ANOVA)).

Supplementary Figure 8

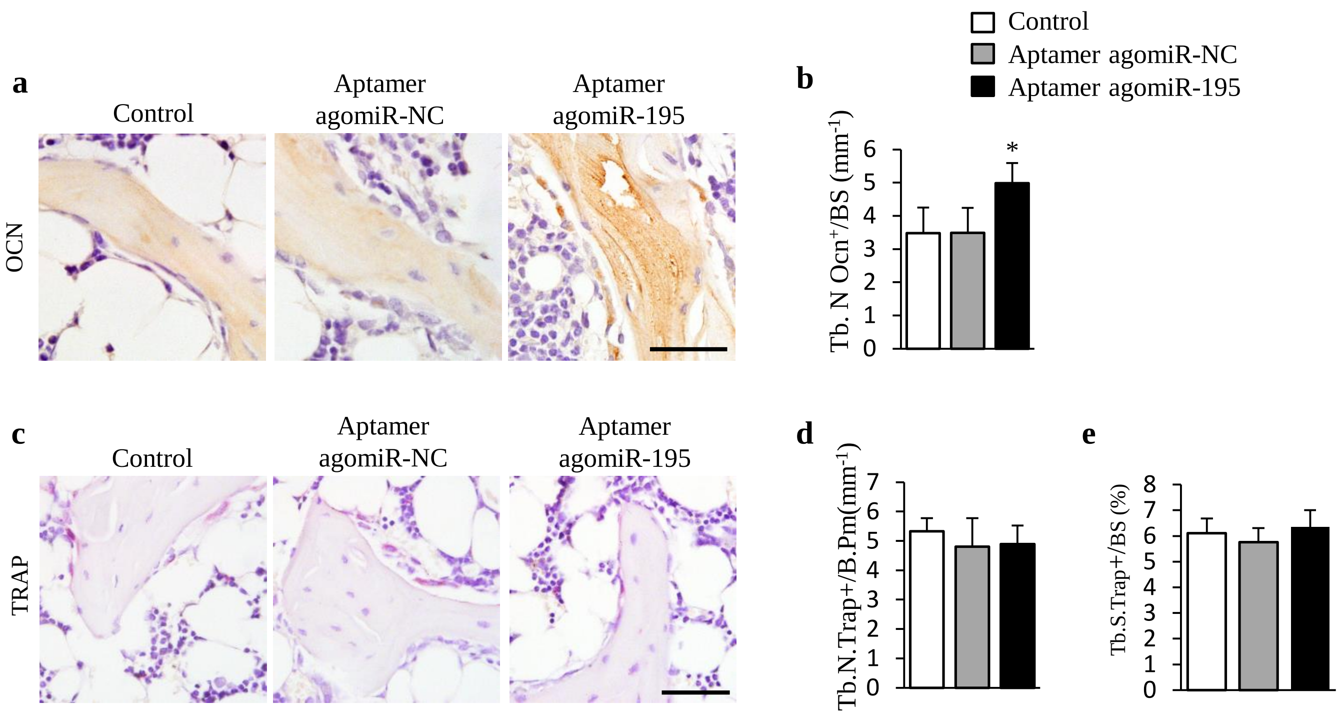

Supplementary Figure 8. Injection of aptamer-agomiR-195 increases osteoblast but not osteoclast number in aged mice.

(**a**) Representative images of osteocalcin immunohistochemical staining with (**b**) quantification of number of osteoblasts in distal femora from aptamer-agomiR-195 treated mice and their controls. (**c**) Representative images of TRAP staining of femora from different time point aptamer-agomiR-195 treated mice and their controls. (**d-e**) Quantification data of $TRAP^+$ cells in trabecular bone surface. Number of $TRAP^+$ cells per bone perimeter ($Tb.N.Trap^+/B.Pm$), and $TRAP^+$ cells surface per bone surface ($Tb.S.Trap^+/BS$) were measured. Scale bar: 50 μm**.** n=5 in each group from three independent experiments. Data shown as mean ± SD. *$P < 0.05$ (analysis of variance (ANOVA)).

Supplementary Figure 9. Uncropped scans of blots

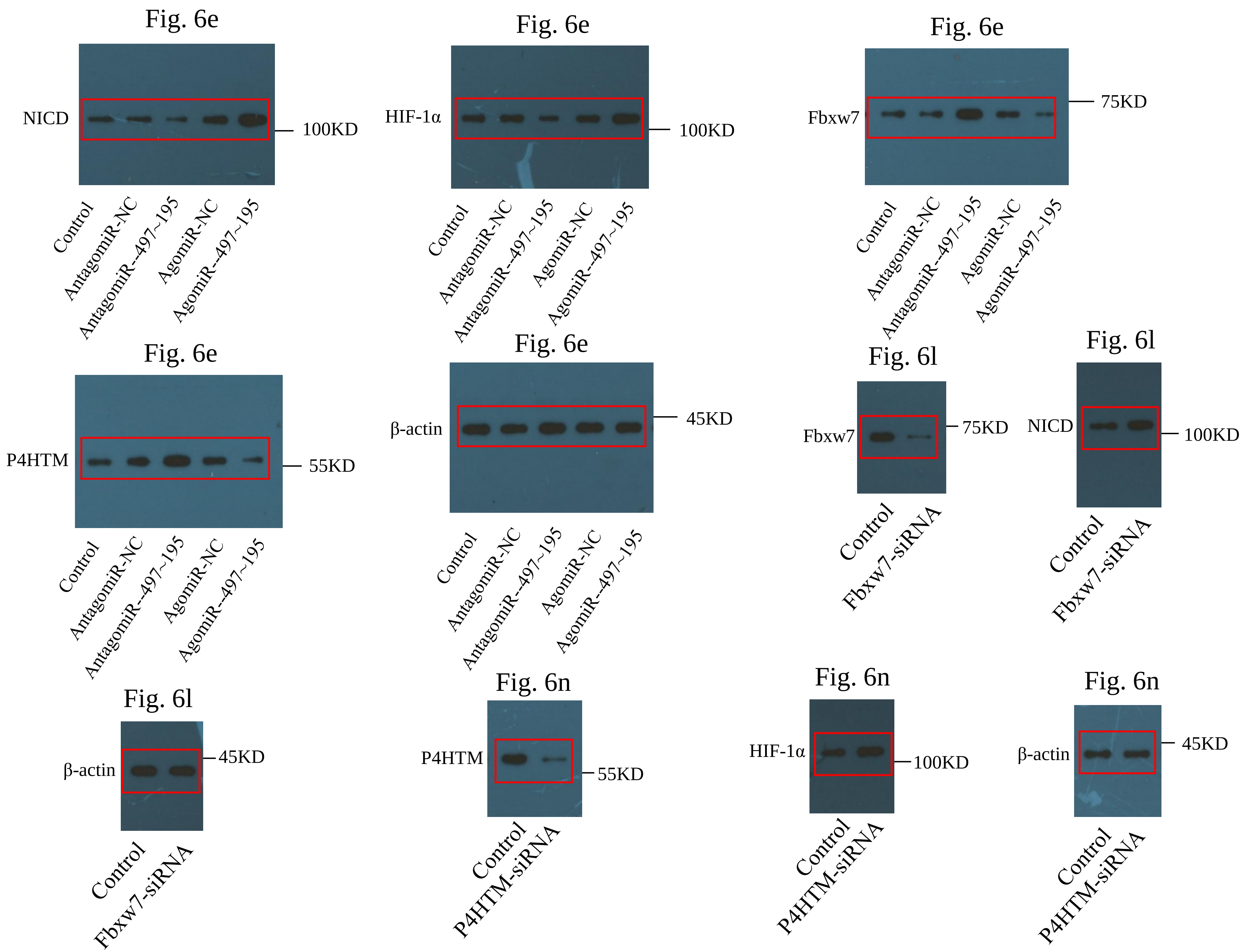

Supplementary Figure 10. Uncropped scans of blots

Supplementary Fig 6 e

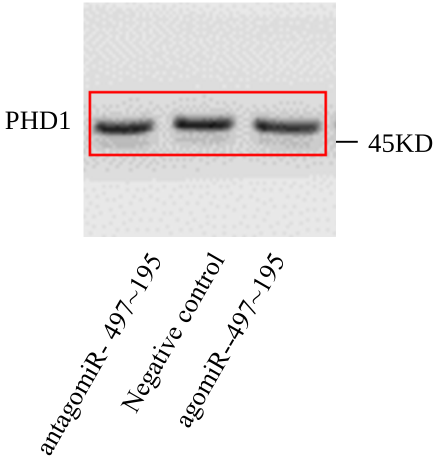

Supplementary Fig 6 e

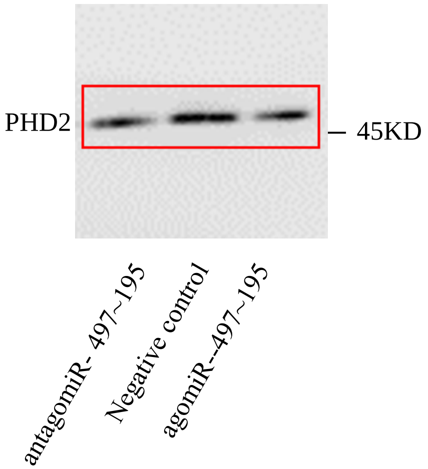

Supplementary Fig 6 e

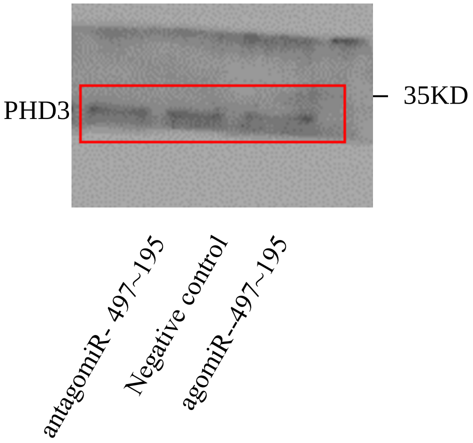

Supplementary Fig 6 e

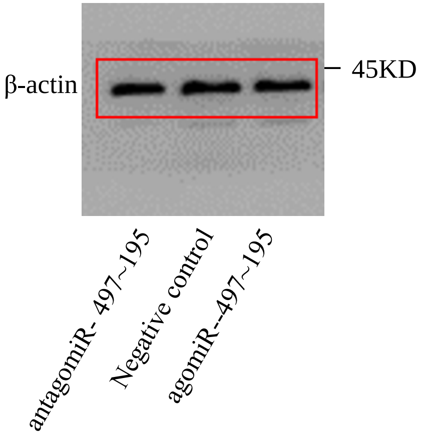

Supplementary Table 1. Nucleotide sequences of primers used for quantitative RT-PCR detection for mRNA

Primers	Forward	Reverse
CD31	CTGCCAGTCCGAAAATGGAAC	CTTCATCCACCGGGGCTATC
Emcn	AATACCAGGCATCGTGTCAGT	CTGATTCTCAGTCTTGTTCTGGG
Notch1	GATGGCCTCAATGGGTACAAG	TCGTTGTTGTTGATGTCACAGT
HIF-1α	ACCTTCATCGGAAACTCCAAAG	CTGTTAGGCTGGGAAAAGTTAGG
Fbxw7	GTTCCGCTGCCTAATCTTCCT	CCCTTCAGGGATTCTGTGCC
P4HTM	ACTCGCCTGGGAAATGGAC	TCCTCATGTACTTGTGGAAGTCT
GAPDH	AGGTCGGTGTGAACGGATTTG	TGTAGACCATGTAGTTGAGGTCA

Supplementary Table 2. Nucleotide sequences of primers used for quantitative RT-PCR detection for microRNA

	Primer	Primer sequence(5' to 3')
miR-195a	RT primer	GTCGTATCCAGTGCAGGGTCCGAGGTATT CGCACTGGATACGACGCCAAT
	Forward	CGTAGCAGCACAGAAAT
	Reverse	GTGCAGGGTCCGAGG
miR-497a	RT primer	GTCGTATCCAGTGCAGGGTCCGAGGTATT CGCACTGGATACGACACAAAC
	Forward	GTGCAGGGTCCGAGGT
	Reverse	TAGCCTGCAGCACACTGTGGT
U6	RT primer	GAACGCTTCACGAATTTGCGTGTCAT
	Forward	CTCGCTTCGGCAGCACA
	Reverse	AACGCTTCACGAATTTGCGT
