# Supplementary figures and images for: Author Correction: MiR-497∼195 cluster regulates angiogenesis during coupling with osteogenesis by maintaining endothelial Notch and HIF-1α activity
Source: Nat Commun. 2025 Jun 23;16:5365. doi: 10.1038/s41467-025-60624-5 (PMC12185676; doi:10.1038/s41467-025-60624-5)

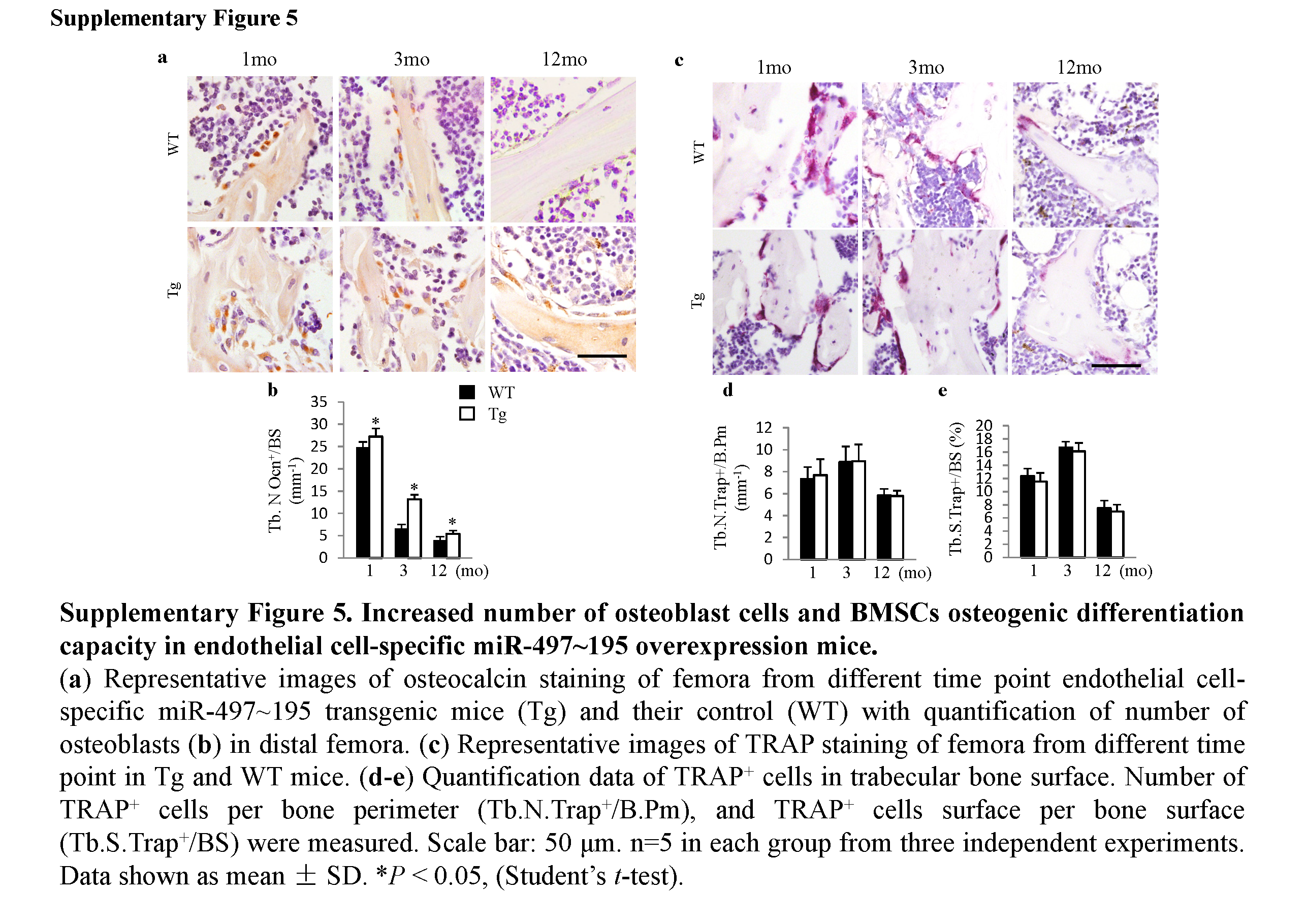

Supplement: Supplementary file 1 — Corrected Supplementary Fig. 5 [file 41467_2025_60624_MOESM1_ESM.tiff]
